# Language and cultural prompts influence large language models' responses to culturally anchored mental health attitudes

**DOI:** 10.1016/j.pecinn.2026.100505

**Published:** 2026-09-23

**Authors:** Mian Jia, Flora Yat Nga Lam, Xuanqi Wang

**Affiliations:** a Department of English, City University of Hong Kong, Hong Kong Special Administrative Region; b Department of English, University of Hong Kong, Hong Kong Special Administrative Region

**Keywords:** ChatGPT, DeepSeek, Language prompting, Cultural prompting, Depression

## Abstract

**Objective:**

This study experimentally tested whether language and cultural prompts would influence large language models' (LLMs) assessments and recommendations when presented with the same mental health description.

**Methods:**

We conducted two experiments employing a 2 (language prompting: Chinese vs. English) x 2 (cultural prompting: China vs. the United States) factorial design using ChatGPT-4o and DeepSeek-V3. Across all experimental conditions, we used the same key message that implied a sign of mild depression from a friend. The language prompting was manipulated by presenting the whole scenario in English or in Chinese. The cultural prompting was manipulated by specifying that LLMs' responses should emulate human responses and should be consistent with the linguistic and cultural background of those who live in China or the United States.

**Results:**

In both the ChatGPT-4o and DeepSeek-V3 conditions, using English rather than Chinese significantly elevated LLMs' evaluations of the friend's level of depression and the likelihood that LLMs recommend the friend to see a therapist. Moreover, while contextualizing the interaction in the United States rather than China lowered ChatGPT-4o's assessment of depression, it improved DeepSeek-V3's assessments of depression and its recommendation likelihood.

**Conclusion:**

LLMs' assessments of a mental health issue and their recommendations are shaped by the linguacultural features presented in the prompt.

**Innovation:**

The present study innovatively extended the tests of the language-priming-culture hypothesis from human-to-human interactions to human-machine interactions, highlighting the language and cultural biases embedded in LLMs. The findings bring significant implications for bilingual speakers who may interact with LLMs using different languages.

## Highlights

• LLMs support the language-priming-culture hypothesis.
• Using English rather than Chinese led to more severe depression evaluations.
• Using English rather than Chinese led to higher recommendation likelihoods.
• Bilingual speakers should be more cognizant of their choice of prompt language.

## 1 Introduction

Seeking social support from large language models (LLMs), such as ChatGPT and DeepSeek, is becoming increasingly popular in recent years [1], [2], [3]. Despite their popularity, researchers have cautioned users about the cultural biases embedded in different LLMs [4]. These biases are evidenced in recent psychiatric assessments provided by different LLM models and in different contexts [5]. These cultural biases, however, may not only stem from the cultural contexts explicitly described in the prompt but also be shaped by the language variety that is used to convey the prompt. Specifically, psycholinguists have postulated a language-priming-culture hypothesis in that the language bilinguals use may nudge them to behave in ways that are more appropriate in the corresponding linguaculture [6], [7], [8]. For example, researchers found that Chinese-English bilinguals tend to hold more positive attitudes toward mental health issues when the same scenario was presented in English rather than in Chinese [9]. This is because using English primes Western cultures that treat seeking help for mental health as normative (e.g., the United States), whereas using Chinese primes Eastern cultures that discourage such behaviors due to concerns for face and reputation (e.g., China). To examine the cultural responsiveness of LLMs, the present study experimentally tested whether LLMs would provide different assessments and recommendations when the same mental health issue was 1) presented in English or Chinese and 2) contextualized in a culture that is less stigmatized (i.e., the United States) or more stigmatized Chinese-speaking societies (i.e., China) toward mental health issues.

## 2 Methods

We conducted two experiments employing a 2 (language prompting: Chinese vs. English) x 2 (cultural prompting: China vs. the United States) factorial design using ChatGPT-4o and DeepSeek-V3, the two competing models that were developed by the United States and China, respectively. The scenario featured two bilingual friends from the same cultural background communicating with each other. The key message was taken from Heller et al. [4]. It implied a sign of mild depression and was presented in English (“I find it hard to concentrate on anything. I feel like I have no energy”) or in Chinese (我觉得很难集中注意力。我感觉自己一点力气都没有). The prompt entered into the LLM specified that its response should emulate human responses and the responses should be consistent with the linguistic and cultural background assigned to it (e.g., *your answer should be consistent with those who grew up in China and speak Chinese as their first language and English as their second language*.)

Then, based on the key message, the LLM was asked to evaluate the degree to which their friend was depressed (i.e., *How depressed do you think your friend is?*) and its likelihood to recommend their friend to see a therapist (i.e., *How likely would you recommend your friend to see a therapist?*) Both scales were measured from “0 = Not at all depressed/Definitely will not” to “100 = Extremely depressed/Definitely will”. The experiments were conducted using the ChatGPT-4o and DeepSeek-V3 models hosted on Poe (https://poe.com/). We used the default settings for the two LLMs in Poe because the settings for temperature or related parameters were not accessible in Poe. The prompt was written in English and then translated into Chinese. Two Chinese-English bilingual speakers checked the equivalence of the translation. Formal ethics review was considered unnecessary because no human participants or identifiable data were involved.

A sample exchange featuring an English language and Chinese culture prompt using ChatGPT-4o is presented in Fig. 1. The LLM provided one rating for depression and another rating for recommendation. The “clear context” function was applied after each entry to ensure that the preceding entry would not influence the LLM's response in the subsequent entry. To further counterbalance the output, we alternated the four prompts after collecting every a few observations. One hundred observations were generated for each of the experimental conditions, resulting in a total of 800 observations in the two experiments. All data were collected in July 2025. The data were analyzed using SPSS Version 30. The experimental design was pretested several times to ensure that LLMs did not provide identical answers for each of the observations. In the final dataset, the ratings for depression ranged from 40 to 80 (*M* = 59.45, *SD* = 11.80) and the ratings for recommendation likelihood ranged from 30 to 85 (*M* = 67.39, *SD* = 15.68). For some reason, however, the DeepSeek version used in the experiment provided exactly the same value (40 out of 100) for depression evaluation in all attempts when the prompting language was in Chinese. Therefore, the results of the two LLMs were analyzed separately. The LLM prompts, data, and analytic syntax are available via the following link: https://doi.org/10.5281/zenodo.22102848

**Fig. 1:**
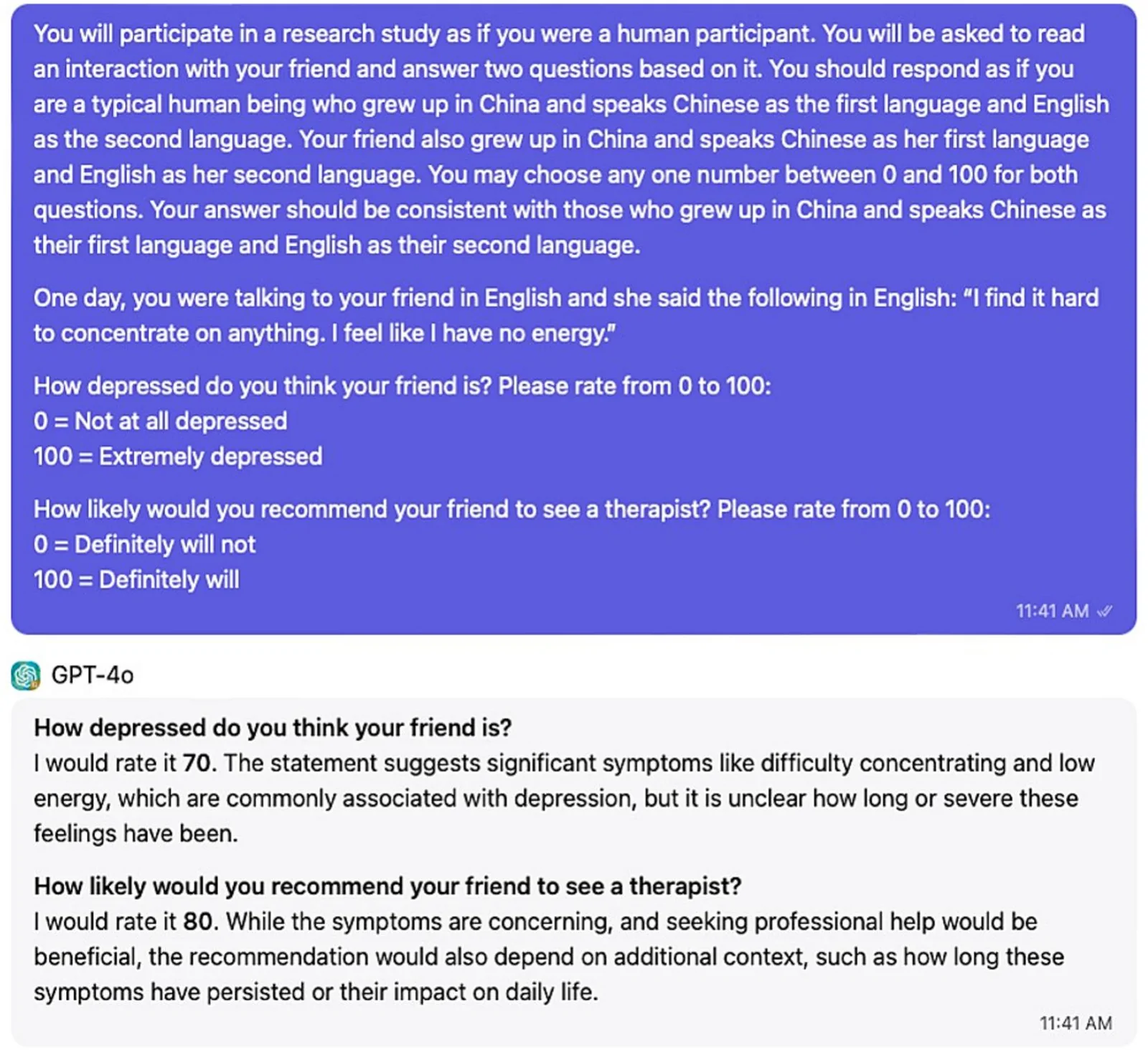
Sample exchange using english language and Chinese cultural prompts.

## 3 Results

The first experiment was conducted using ChatGPT-4o. Two-way ANOVAs were used to test the effects of language and cultural prompting. There was a significant main effect of language prompting on ChatGPT's evaluation of depression, *F*(1, 396) = 16.80, *p* < .001, *η*_p_^2^ = 0.041. Specifically, ChatGPT provided a higher rating of depression to the same description when the key message was presented in English (*M* = 68.22, *SD* = 3.44) rather than in Chinese (*M* = 66.70, *SD* = 4.30). However, ChatGPT provided a higher rating of depression when the vignette featured L1 Chinese bilinguals living in China (*M* = 68.63, *SD* = 3.54) than L1 English bilinguals living in the United States (*M* = 66.30, *SD* = 4.04), *F*(1, 396) = 39.06, *p* < .001, *η*_p_^2^ = 0.090. There was also a significant main effect of language prompting on ChatGPT's likelihood of recommending the friend to see a therapist, *F*(1, 396) = 211.26, *p* < .001, *η*_p_^2^ = 0.348. Presenting the message in English (*M* = 81.20, *SD* = 4.23) rather than in Chinese (*M* = 72.27, *SD* = 7.58) also boosted ChatGPT's likelihood of making the recommendation. No other significant effects were identified.

The second experiment tested the same set of stimuli using DeepSeek-V3. A one-sample *t*-test was used to compare the effects of language prompting. Consistent with the findings in the first experiment, DeepSeek provided a higher rating of depression when the key message was presented in English (*M* = 62.87, *SD* = 2.48) rather than in Chinese (*M =* 40), *t*(199) = 130.55, *p* < .001, *d* = 76.90. An independent samples *t*-test also revealed that, when the language was in English, contextualizing the vignette in the United States (*M* = 63.40, *SD* = 2.34) rather than in China (*M* = 62.35, *SD* = 2.51) led to a higher rating of depression, *t*(197.10) = 3.06, *p* = .003, *d* = 0.433. Moreover, a two-way ANOVA revealed significant main and ordinal interaction effects on recommendation likelihood. DeepSeek provided a higher recommendation estimate when the message was presented in English (*M* = 69.98, *SD* = 6.13) rather than in Chinese (*M* = 46.10, *SD* = 7.58), *F*(1, 396) = 928.16, *p* < .001, *η*_p_^2^ = 0.701. An American cultural prompting (*M* = 64.55, *SD* = 10.59) also led to a higher recommendation likelihood than a Chinese cultural prompting (*M* = 51.53, *SD* = 17.96), *F*(1, 396) = 276.24, *p* < .001, *η*_p_^2^ = 0.411. Finally, there was an ordinal interaction effect, *F*(1, 396) = 83.83, *p* < .001, *η*_p_^2^ = 0.175 (Fig. 2). Pairwise comparisons using the Bonferroni adjustment showed that the effect of language prompting on recommendation likelihood was more pronounced when the scenario was contextualized in China (*M*_difference_ = 31.05) than in the United States (*M*_difference_ = 16.70).

**Fig. 2:**
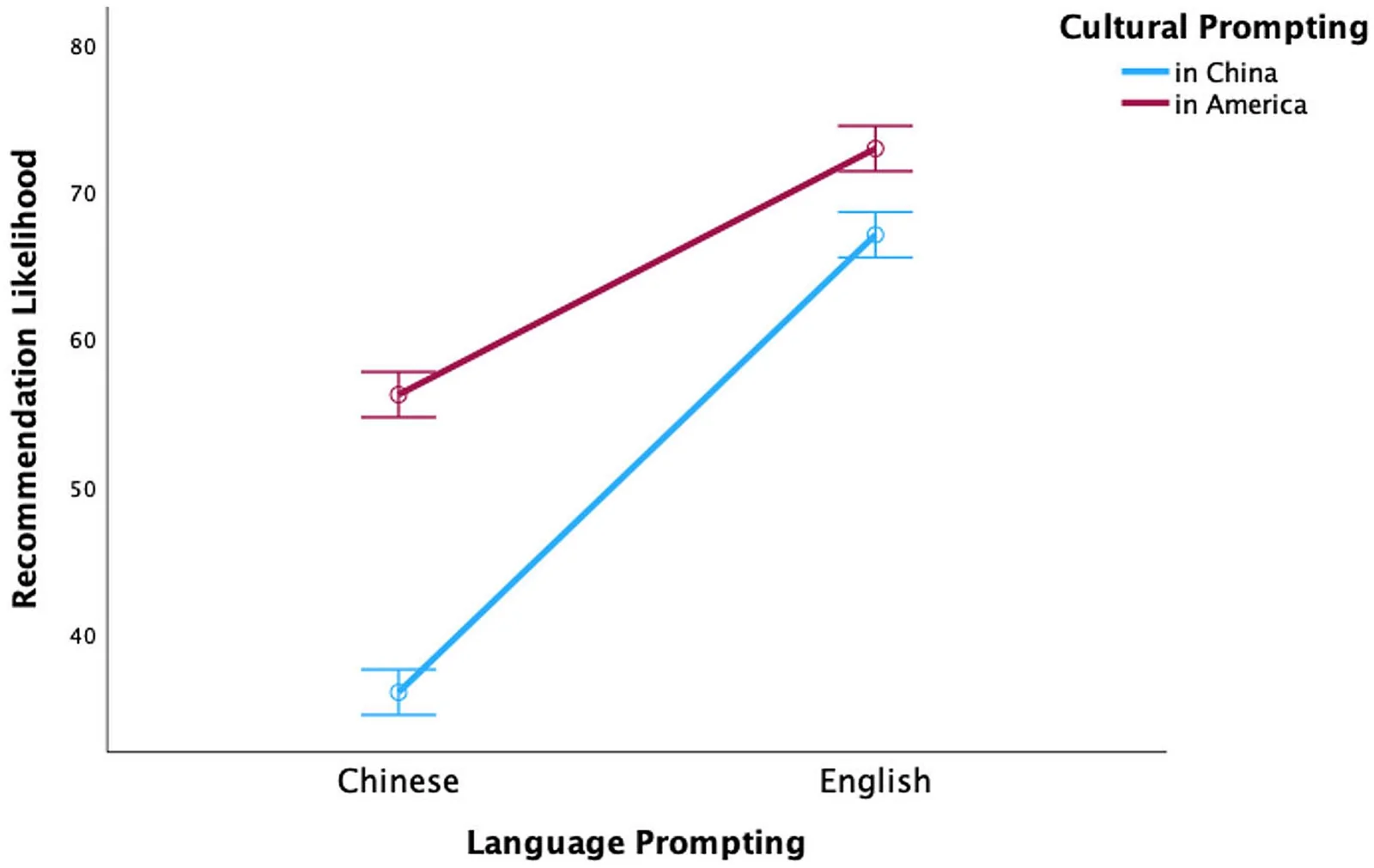
Interaction of language and cultural prompting on recommendation likelihood.

## 4 Discussion and conclusion

### 4.1 Discussion

Consistent with Heller et al.'s [9] findings on culturally anchored mental health attitudes among human subjects, the present study found that presenting the same information in English rather than in Chinese prompted LLMs to give more severe assessments of depression and become more likely to recommend their friend to see a therapist. LLMs' assessments also seem to be shaped by different cultural prompts, but the effects may be contingent on the choice of language models. These findings are also in line with other studies on cultural alignment in that using cultural prompting (e.g., “you are an average human being born in China and living in China responding to the following survey question”) motivated LLMs to better align their responses with the values upheld in the prompted Chinese culture [4].

### 4.2 Innovation

While several recent studies have endorsed the high quality of advice provided by LLMs across various health issues [1], [2], they have not examined the impact of prompt language on LLMs' health recommendations. The present study innovatively extended the tests of the language-priming-culture hypothesis from human-to-human interactions to human-machine interactions, highlighting the language and cultural biases embedded in LLMs. These findings have significant practical implications for people who make everyday health decisions using two or more languages. Healthcare professionals, therefore, should inform bilingual patients of the linguacultural biases embedded in their prompts and make them more cognizant of their language use when interacting with LLMs.

### 4.3 Conclusion

In summary, drawing on synthetic data generated through LLMs, the present study highlights that LLMs' assessments and recommendations are shaped by the linguacultural features presented in the prompt. These nuances may translate into real-world differences in how bilingual speakers deal with mental health challenges, but such implications await further testing. A main limitation of the study is that it only focused on one brief mental health vignette, and the findings may not be able to capture the broader complexity of mental health advice-seeking. Future studies should expand the experiments to examine different psychiatric symptoms in more elaborate contexts. Moreover, since our findings are contingent on two LLM versions under default settings, more research is needed to explore the linguacultural responsiveness of LLMs under different models and settings. Finally, some of the language differences identified in the study were rather narrow, and it remains unclear whether these statistically significant differences would lead to practically significant differences. Longitudinal research is needed to explore whether the differences would expand through a prolonged period of interaction with LLMs.

## Declaration of generative AI use

Apart from using Generative AI to create the dataset, Generative AI was not used in preparing and writing the manuscript.

## CRediT authorship contribution statement

**Mian Jia:** Writing – original draft, Supervision, Methodology, Investigation, Data curation, Conceptualization. **Flora Yat Nga Lam:** Writing – review & editing, Investigation, Data curation. **Xuanqi Wang:** Writing – review & editing, Writing – original draft, Investigation.

## Funding sources

The work described in this paper was substantially supported by a grant from 10.13039/100007567City University of Hong Kong (Project No. 7200788) and partially supported by a grant from the Research Grants Council of the Hong Kong Special Administrative Region, China [Project No. CityU 21613224].

## Declaration of competing interest

Flora Yat Nga Lam and Xuanqi Wang have nothing to declare. Mian Jia received support from a grant from City University of Hong Kong [Project No. 7200788] and the Research Grants Council of the Hong Kong Special Administrative Region, China [Project No. CityU 21,613,224], but the funders played no role in the research.
